# Crystal structure of borated *N*,*N*,*N*′,*N*′-tetra­methyldi­amino­methane

**DOI:** 10.1107/S2056989015016813

**Published:** 2015-09-12

**Authors:** Kathrin Louven, Georgina Quentin, Carsten Strohmann

**Affiliations:** a Fakultät für Chemie und Chemische Biologie, Technische Universität Dortmund, Otto-Hahn-Strasse 6, 44221 Dortmund, Germany

**Keywords:** crystal structure, borane, amine, twin

## Abstract

In the title compound, {[(di­methyl­amino)­meth­yl]di­methyl­amine}­trihydridoboron, C_5_H_17_BN_2_, the tetra­hedral geometry of the N—C—N unit is slightly disorted. As a result of the bulky amine substituents, a wider N—C—N angle of 113.6 (1)° is observed. The bond lengths between the N atom and methyl groups are slighly elongated to 1.481 (2) and 1.482 (2) Å at the borated N atom, whereas the distances between the other N atom and its methyl groups are only 1.461 (2) and 1.462 (2) Å. The studied crystal was twinned. The twin data refinement was subsequently carried out with a scale factor of 0.263 (1). The two lattices of the twin domains were rotated by 179.84°.

## Related literature   

For background to boranes, see: Falbe & Regitz (1999 ▸). Burg & Schlesinger (1937 ▸) reported the first borane amine complex. A feature of boranes is their metal character and pronounced Lewis acidity (Huheey *et al.*, 1995 ▸). This Lewis acidity is used to enable the α-deprotonation of tertiary amines (Kessar *et al.*, 1991 ▸; Ebden *et al.*, 1995 ▸). Our group frequently uses methods to deprotonate compounds in α-position (Strohmann & Gessner, 2007 ▸; Gessner & Strohmann, 2012 ▸). For crystal structures containing the borated *N*,*N*,*N*′,*N*′-tetra­methyldi­amino­methane motif, see: Fang *et al.* (1994 ▸); Hanic & Šubrtová (1969 ▸); Flores-Parra *et al.* (1999 ▸); Rojas-Lima *et al.* (2000 ▸). For comparison with other structures with di­methyl­amino­borane moiety, see: Gollas *et al.* (2013 ▸); Bera *et al.* (2011 ▸); Ramachandran *et al.* (2004 ▸); Netz *et al.* (2005 ▸). For diborated tetra­methyl­ethylenedi­amine, see: Chitsaz *et al.* (2001 ▸).
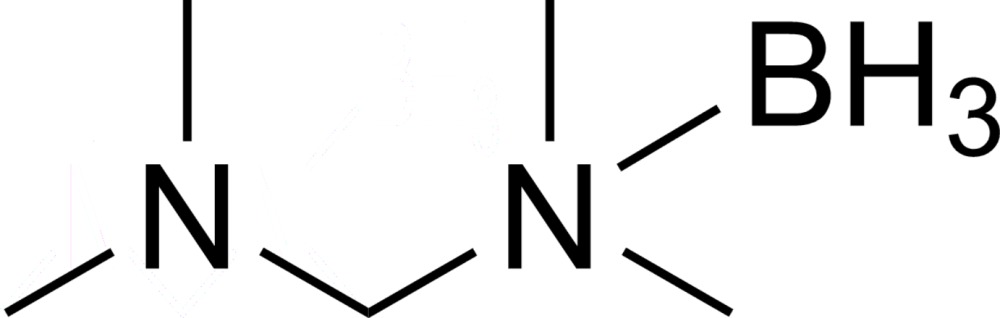


## Experimental   

### Crystal data  

C_5_H_17_BN_2_
*M* *_r_* = 116.01
Triclinic, 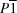

*a* = 6.0464 (8) Å
*b* = 7.6987 (9) Å
*c* = 9.5896 (11) Å
α = 69.602 (10)°
β = 76.519 (11)°
γ = 74.912 (10)°
*V* = 398.95 (9) Å^3^
*Z* = 2
Mo *K*α radiation
μ = 0.06 mm^−1^
*T* = 173 K
0.2 × 0.15 × 0.15 mm

### Data collection  

AgilentXcalibur, Sapphire3 diffractometer
Absorption correction: multi-scan (*CrysAlis PRO*; Agilent, 2014 ▸) *T*_min_ = 0.983, *T*_max_ = 1.000
3232 measured reflections
3232 independent reflections
1828 reflections with *I* > 2σ(*I*)

### Refinement  

*R*[*F* ^2^ > 2σ(*F* ^2^)] = 0.041
*wR*(*F* ^2^) = 0.099
*S* = 0.87
3232 reflections
90 parameters
H atoms treated by a mixture of independent and constrained refinement
Δρ_max_ = 0.18 e Å^−3^
Δρ_min_ = −0.22 e Å^−3^

### 

Data collection: *CrysAlis PRO* (Agilent, 2014 ▸); cell refinement: *CrysAlis PRO*; data reduction: *CrysAlis PRO*; program(s) used to solve structure: *SHELXS96* (Sheldrick, 2008 ▸); program(s) used to refine structure: *SHELXL96* (Sheldrick, 2008 ▸); molecular graphics: *SHELXTL* (Sheldrick, 2008 ▸); software used to prepare material for publication: *OLEX2* (Dolomanov *et al.*, 2009 ▸).

## Supplementary Material

Crystal structure: contains datablock(s) I. DOI: 10.1107/S2056989015016813/zq2234sup1.cif

Structure factors: contains datablock(s) I. DOI: 10.1107/S2056989015016813/zq2234Isup2.hkl

**Fig. 1.**
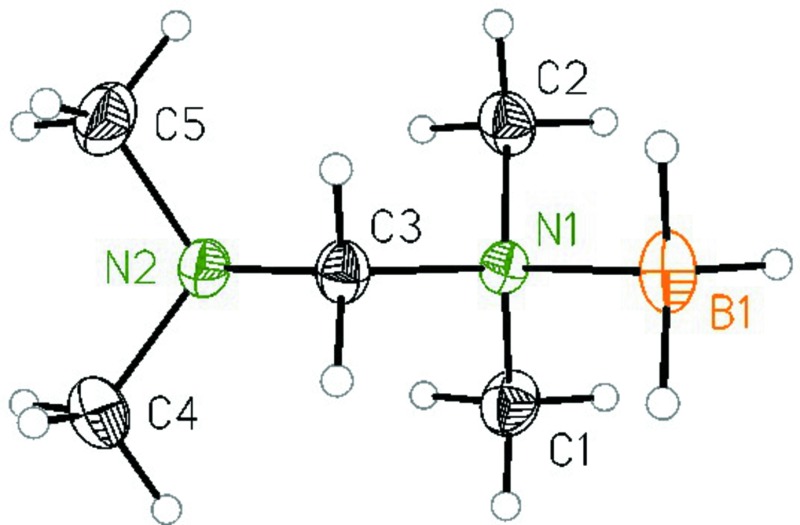
Molecular structure of the title compound with displacement ellipsoids drawn at 50% probability level.

**Fig. 2.**
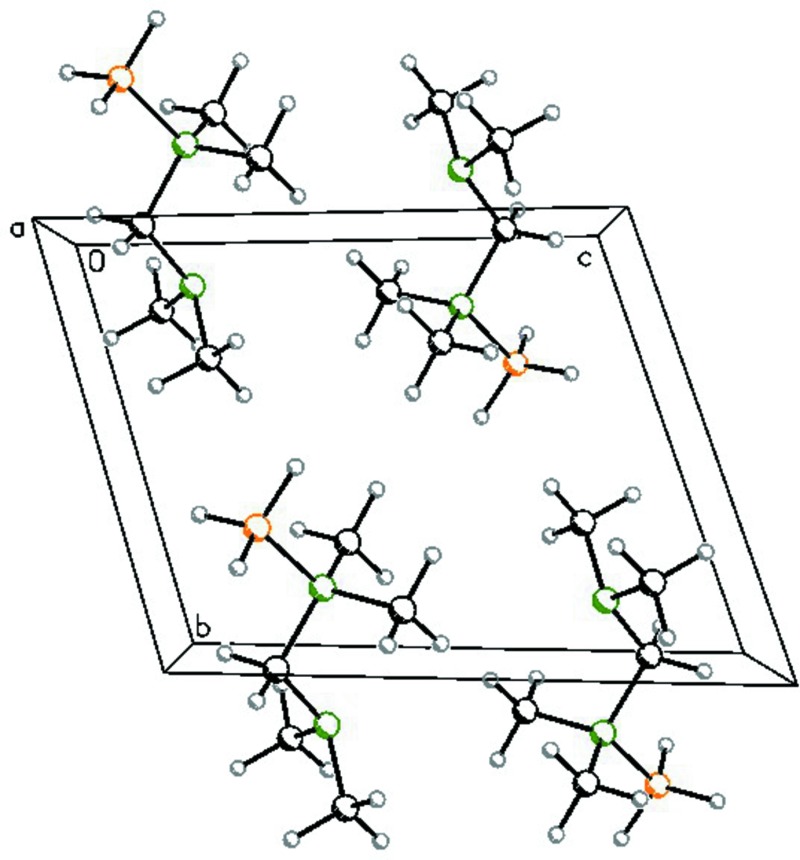
Molecular packing viewed along the a axis.

. DOI: 10.1107/S2056989015016813/zq2234fig1.tif
Mol­ecular structure of the title compound with displacement ellipsoids drawn at 50% probability level.

a . DOI: 10.1107/S2056989015016813/zq2234fig2.tif
Mol­ecular packing viewed along the *a* axis.

CCDC reference: 1422998

Additional supporting information:  crystallographic information; 3D view; checkCIF report

## supplementary crystallographic information

### S1. Comment

Boranes are a useful substance in today's chemistry. In 1979, Herbert C. Brown received the Nobel Prize in chemistry for his studies on these inter­esting compounds (Falbe & Regitz, 1999). Special about boranes is their metal character and pronounced Lewis acidity (Huheey *et al.*, 1995). This Lewis acidity is used to enable the α-deprotonation of tertiary amines (Kessar *et al.*, 1991; Ebden *et al.*, 1995). Our group frequently uses methods to deprotonate compounds in α-position (Gessner & Strohmann, 2007; Gessner & Strohmann, 2012). Here, BH_3_ was added to *N*,*N,N',N'*-tetra­methyldi­amino­methane (TMMDA) in order to deplete the nitro­gen's +M-effect, smoothing the way for α-li­thia­tion. We isolated and structurally characterized the borated TMMDA for the first time. Li­thia­tion of the product however was not successful.

The title compound crystallizes in the triclinic crystal system with space group P-1. The N–C–N-bonds are not equidistant. Longer N–C-bonds are observed for the borated nitro­gen [N1–C1 1.4806 (17) Å, N1–C2 1.4818 (17) Å, N1–C3 1.5039 (16) Å] than for the other [N2–C3 1.4393 (17) Å, N2–C4 1.4612 (18) Å, N2–C5 1.4622 (18) Å]. Furthermore, bond angles at the nitro­gen atoms differ. Due to steric hindrance of the methyl groups, angles on N2 are found to be broader [C3–N2–C4 112.82 (11)°, C3–N2–C5 112.85 (12)°, C4–N2–C5 110.31 (11)°] than the ideal sp^3^-angle of 109.5°. A torsion angle of 179.82 (13)° can be observed for B1–N1–C3–N2, placing B1 as far away from C3 and its hydrogen atoms as possible.

### S1.1. Experimental

BH_3_-solution (11.7 mL, 1 M in thf, 11.7 mmol) was added to *N,N,N',N'*-tetra­methyldi­amino­methane (9.79 mmol, 1.00 g) at 0 °C. The reaction mixture was stirred for 3 h at room temperature after which saturated K_2_CO_3_-solution (7 mL) was added. The reaction was allowed to continue for 72 h at room temperature. After extraction with Et_2_O (3x10 mL), the combined extracts were dried (Na_2_SO_4_). After evaporation under reduced pressure, colorless crystals precipitated (694 mg, 5.98 mmol, 61% yield).

### S1.2. Refinement

Crystal data, data collection and structure refinement details are summarized in Table 1. Hydrogen atoms linked to carbon were placed and refined by using the riding model (C–H = 0.95-0.99 Å, *U*_iso_(H) = 1.2 *U*_eq_(C) and *U*_iso_(H) = 1.5 *U*_eq_(C) for terminal groups). Hydrogen atoms linked to boron were taken from difference Fourier maps.

Twin domains were found in the crystal and refined to a ratio of 0.26/0.74. The two lattices were rotated by 179.84°. HKLF5 refletion file was used for refinement.

### Crystal data

**Table:**

C_5_H_17_BN_2_	*Z* = 2
*M**_r_* = 116.01	*F*(000) = 132
Triclinic, *P*1	*D*_x_ = 0.966 Mg m^−^^3^
*a* = 6.0464 (8) Å	Mo *K*α radiation, λ = 0.71073 Å
*b* = 7.6987 (9) Å	Cell parameters from 1858 reflections
*c* = 9.5896 (11) Å	θ = 3.0–28.3°
α = 69.602 (10)°	µ = 0.06 mm^−^^1^
β = 76.519 (11)°	*T* = 173 K
γ = 74.912 (10)°	Block, clear light colourless
*V* = 398.95 (9) Å^3^	0.2 × 0.15 × 0.15 mm

### Data collection

**Table:**

AgilentXcalibur, Sapphire3 diffractometer	3232 measured reflections
Radiation source: Enhance (Mo) X-ray Source	3232 independent reflections
Graphite monochromator	1828 reflections with *I* > 2σ(*I*)
Detector resolution: 16.0560 pixels mm^-1^	θ_max_ = 27.0°, θ_min_ = 3.1°
ω scans	*h* = −7→7
Absorption correction: multi-scan (*CrysAlis PRO*; Agilent, 2014)	*k* = −9→9
*T*_min_ = 0.983, *T*_max_ = 1.000	*l* = −12→12

### Refinement

**Table:**

Refinement on *F*^2^	Primary atom site location: structure-invariant direct methods
Least-squares matrix: full	Hydrogen site location: mixed
*R*[*F*^2^ > 2σ(*F*^2^)] = 0.041	H atoms treated by a mixture of independent and constrained refinement
*wR*(*F*^2^) = 0.099	*w* = 1/[σ^2^(*F*_o_^2^) + (0.0498*P*)^2^] where *P* = (*F*_o_^2^ + 2*F*_c_^2^)/3
*S* = 0.87	(Δ/σ)_max_ = 0.001
3232 reflections	Δρ_max_ = 0.18 e Å^−^^3^
90 parameters	Δρ_min_ = −0.22 e Å^−^^3^
0 restraints	

### Special details

**Table:**

Geometry. All e.s.d.'s (except the e.s.d. in the dihedral angle between two l.s. planes) are estimated using the full covariance matrix. The cell e.s.d.'s are taken into account individually in the estimation of e.s.d.'s in distances, angles and torsion angles; correlations between e.s.d.'s in cell parameters are only used when they are defined by crystal symmetry. An approximate (isotropic) treatment of cell e.s.d.'s is used for estimating e.s.d.'s involving l.s. planes.
Refinement. Refined as a 2-component twin. 1. Twinned data refinement Scales: 0.7367 (11) 0.2633 (11) 2. Fixed *U*_iso_ At 1.2 times of: All C(H,H) groups At 1.5 times of: All C(H,H,H) groups 3.a Secondary CH2 refined with riding coordinates: C3(H3A,H3B) 3.b Idealized Me refined as rotating group: C2(H2A,H2B,H2C), C1(H1A,H1B,H1C), C5(H5A,H5B,H5C), C4(H4A,H4B,H4C)

### Fractional atomic coordinates and isotropic or equivalent isotropic displacement parameters (Å^2^)

**Table:**

	*x*	*y*	*z*	*U*_iso_*/*U*_eq_	
N1	0.17275 (18)	0.17659 (15)	0.69988 (12)	0.0182 (3)	
C3	0.2336 (2)	−0.00477 (18)	0.82279 (15)	0.0214 (3)	
H3A	0.2756	0.0246	0.9048	0.026*	
H3B	0.0951	−0.0634	0.8648	0.026*	
N2	0.42225 (19)	−0.13895 (16)	0.77307 (13)	0.0226 (3)	
C2	0.1062 (3)	0.1356 (2)	0.57701 (16)	0.0274 (4)	
H2A	0.2389	0.0568	0.5326	0.041*	
H2B	0.0591	0.2545	0.4995	0.041*	
H2C	−0.0233	0.0685	0.6177	0.041*	
C1	0.3751 (2)	0.2702 (2)	0.63738 (18)	0.0293 (4)	
H1A	0.4230	0.2959	0.7179	0.044*	
H1B	0.3328	0.3894	0.5589	0.044*	
H1C	0.5036	0.1873	0.5939	0.044*	
C5	0.3510 (3)	−0.3102 (2)	0.78120 (19)	0.0340 (4)	
H5A	0.2924	−0.3745	0.8861	0.051*	
H5B	0.4844	−0.3946	0.7432	0.051*	
H5C	0.2284	−0.2762	0.7197	0.051*	
B1	−0.0405 (3)	0.3123 (3)	0.7713 (2)	0.0289 (4)	
C4	0.6200 (3)	−0.1878 (2)	0.85213 (18)	0.0333 (4)	
H4A	0.6693	−0.0721	0.8429	0.050*	
H4B	0.7483	−0.2698	0.8077	0.050*	
H4C	0.5749	−0.2542	0.9588	0.050*	
H1D	−0.083 (2)	0.4427 (19)	0.6758 (15)	0.032 (4)*	
H1E	−0.189 (2)	0.2332 (19)	0.8155 (16)	0.034 (4)*	
H1F	0.023 (2)	0.3383 (19)	0.8634 (17)	0.036 (4)*	

### Atomic displacement parameters (Å^2^)

**Table:**

	*U*^11^	*U*^22^	*U*^33^	*U*^12^	*U*^13^	*U*^23^
N1	0.0196 (6)	0.0158 (6)	0.0184 (6)	−0.0027 (5)	−0.0032 (5)	−0.0047 (5)
C3	0.0245 (8)	0.0191 (7)	0.0183 (7)	−0.0020 (6)	−0.0041 (6)	−0.0041 (6)
N2	0.0222 (7)	0.0187 (6)	0.0260 (7)	−0.0015 (5)	−0.0044 (5)	−0.0072 (5)
C2	0.0311 (9)	0.0279 (9)	0.0229 (8)	0.0000 (7)	−0.0093 (7)	−0.0081 (7)
C1	0.0259 (9)	0.0240 (8)	0.0347 (9)	−0.0071 (7)	0.0007 (7)	−0.0066 (7)
C5	0.0367 (10)	0.0230 (8)	0.0421 (10)	−0.0019 (7)	−0.0055 (8)	−0.0131 (8)
B1	0.0284 (10)	0.0236 (9)	0.0273 (10)	0.0026 (8)	−0.0018 (8)	−0.0063 (8)
C4	0.0255 (9)	0.0317 (9)	0.0404 (10)	0.0024 (7)	−0.0102 (8)	−0.0107 (8)

### Geometric parameters (Å, º)

**Table:**

N1—C3	1.5039 (16)	C1—H1A	0.9800
N1—C2	1.4818 (17)	C1—H1B	0.9800
N1—C1	1.4806 (17)	C1—H1C	0.9800
N1—B1	1.615 (2)	C5—H5A	0.9800
C3—H3A	0.9900	C5—H5B	0.9800
C3—H3B	0.9900	C5—H5C	0.9800
C3—N2	1.4393 (17)	B1—H1D	1.117 (13)
N2—C5	1.4622 (18)	B1—H1E	1.131 (14)
N2—C4	1.4612 (18)	B1—H1F	1.137 (14)
C2—H2A	0.9800	C4—H4A	0.9800
C2—H2B	0.9800	C4—H4B	0.9800
C2—H2C	0.9800	C4—H4C	0.9800
			
C3—N1—B1	108.16 (10)	N1—C1—H1C	109.5
C2—N1—C3	109.62 (10)	H1A—C1—H1B	109.5
C2—N1—B1	110.43 (11)	H1A—C1—H1C	109.5
C1—N1—C3	110.11 (10)	H1B—C1—H1C	109.5
C1—N1—C2	108.59 (10)	N2—C5—H5A	109.5
C1—N1—B1	109.93 (12)	N2—C5—H5B	109.5
N1—C3—H3A	108.8	N2—C5—H5C	109.5
N1—C3—H3B	108.8	H5A—C5—H5B	109.5
H3A—C3—H3B	107.7	H5A—C5—H5C	109.5
N2—C3—N1	113.61 (10)	H5B—C5—H5C	109.5
N2—C3—H3A	108.8	N1—B1—H1D	105.7 (7)
N2—C3—H3B	108.8	N1—B1—H1E	106.1 (7)
C3—N2—C5	112.85 (12)	N1—B1—H1F	106.4 (7)
C3—N2—C4	112.82 (11)	H1D—B1—H1E	111.1 (10)
C4—N2—C5	110.31 (11)	H1D—B1—H1F	113.7 (10)
N1—C2—H2A	109.5	H1E—B1—H1F	113.2 (10)
N1—C2—H2B	109.5	N2—C4—H4A	109.5
N1—C2—H2C	109.5	N2—C4—H4B	109.5
H2A—C2—H2B	109.5	N2—C4—H4C	109.5
H2A—C2—H2C	109.5	H4A—C4—H4B	109.5
H2B—C2—H2C	109.5	H4A—C4—H4C	109.5
N1—C1—H1A	109.5	H4B—C4—H4C	109.5
N1—C1—H1B	109.5		
			
N1—C3—N2—C5	−114.53 (13)	C1—N1—C3—N2	−60.04 (14)
N1—C3—N2—C4	119.61 (12)	B1—N1—C3—N2	179.82 (13)
C2—N1—C3—N2	59.36 (14)		
