# Supplementary figures and images for: Crystal structure of borated N,N,N′,N′-tetra­methyldi­amino­methane
Source: Acta Crystallogr E Crystallogr Commun. 2015 Sep 12;71(Pt 10):o743–4. doi: 10.1107/S2056989015016813 (PMC4647418; doi:10.1107/S2056989015016813)

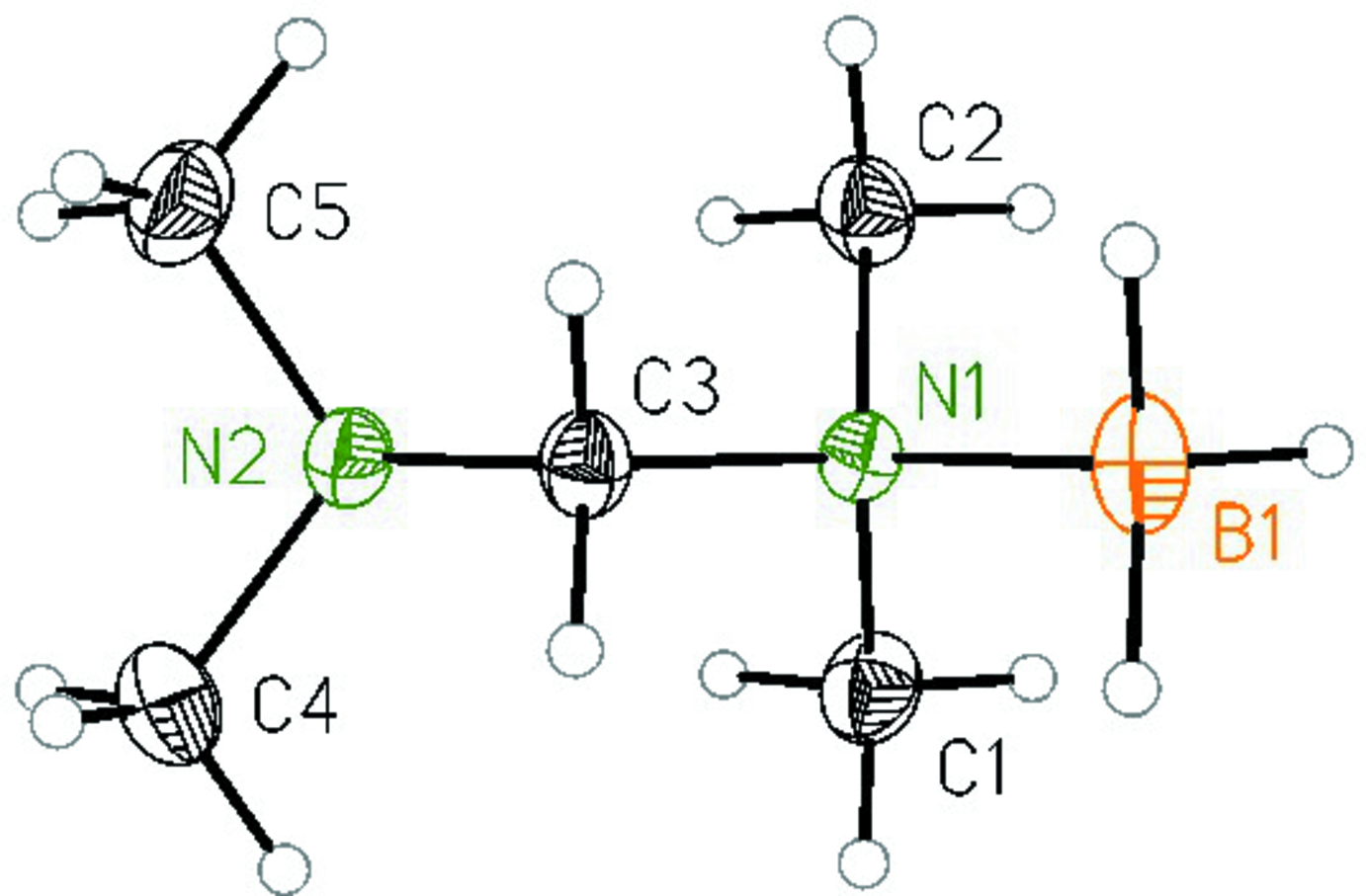

Supplement: Supplementary file 3 [file e-71-0o743-fig1.tif]

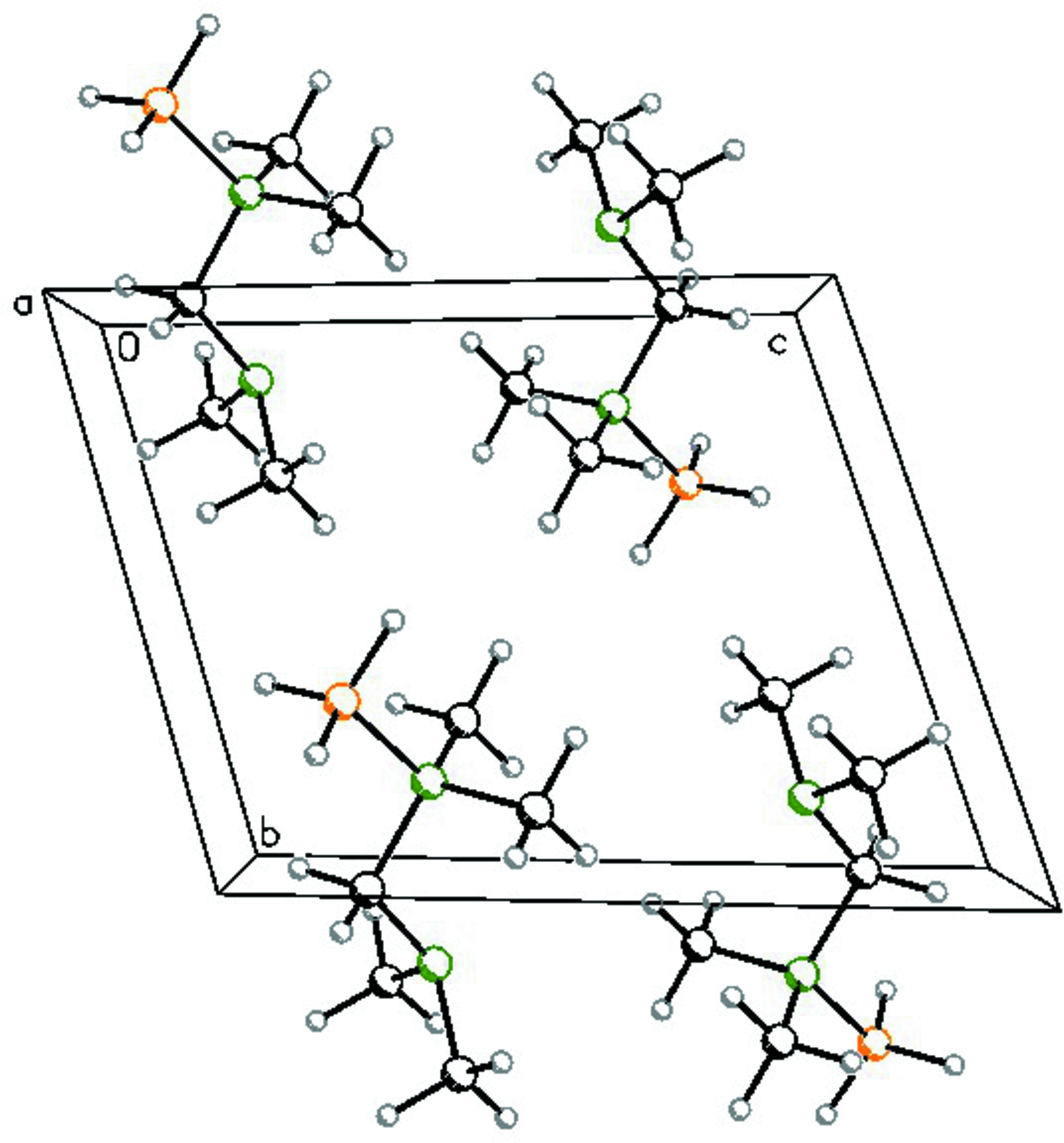

Supplement: Supplementary file 4 [file e-71-0o743-fig2.tif]
